# The Influence of Diet Containing Wheat Gluten Supplemented with Dipeptides or Amino Acids on the Morphology of White Muscle of Yellow Perch (*Perca flavescens*)

**DOI:** 10.3390/ani10030388

**Published:** 2020-02-27

**Authors:** Maciej Kamaszewski, Maciej Wójcik, Agata Krawczyńska, Teresa Ostaszewska

**Affiliations:** 1 Department of Ichthyobiology, Fisheries and Aquaculture Biotechnology, Faculty of Animal Science, Warsaw University of Life Sciences-SGGW (WULS-SGGW), Ciszewskiego 8, 02-786 Warsaw, Poland; maciej_kamaszewski@sggw.pl (M.K.); teresa_ostaszewska@sggw.pl (T.O.); 2 The Kielanowski Institute of Animal Physiology and Nutrition, Polish Academy of Sciences, Instytucka 3, 05-110 Jabłonna, Poland; a.krawczynska@ifzz.pl

**Keywords:** alternative protein source, amino acid, dipeptide, lysine, white muscle, yellow perch

## Abstract

**Simple Summary:**

The research was carried out to determine the possibility of feeding the predatory species using feed formulated with a plant protein source—wheat gluten. Percid fish such as yellow perch, European perch, or pikeperch are more and more often an object of rearing in pond aquaculture and recirculating aquaculture system (RAS). This is particularly important because aquaculture is constantly developing in the world and meets the limited possibilities of using natural resources for fishmeal production. Moreover, fishing for the production of fishmeal adversely affects the natural environment, including the conservation status of predatory fish species which play a key role in maintaining the balance of the aquatic environment. The study showed that the use of feed formulated on wheat gluten is beneficial in yellow perch feeding. Furthermore, feed containing wheat gluten compared to commercial feed based on fish meal showed no significant differences in the growth rate and survival of fish. This is important as a consequence of the implementation of sustainable and nature-friendly aquaculture through the protection of natural resources such as protection of schools of fish being the source of fishmeal. Moreover, over the past 40 years, the price of fishmeal increased significantly and as other products used to produce fish feed are subject to fluctuations. This is caused by many factors, including increased demand, reduction of natural resources, or climate change. Currently, the prices of wheat gluten and fishmeal are at a comparable level. Therefore, it seems that a balanced development of the aquaculture sector will be associated with the protection of natural fish stocks and, therefore, with greater use of agriculture products in animal nutrition.

**Abstract:**

Nutrition affects the metabolism of muscle cells and myogenic progenitor cells which play a crucial role in the growth and development of the muscle tissue. Because of the fact that the development process of yellow perch muscle tissue is not well known, the study aimed to analyze the influence of diets containing wheat gluten and supplemented with Lys and Gly in dipeptides or free form. Fish were allocated into 12 tanks and divided into four groups. Two of the experimental diets were supplemented Lys-Gly in the dipeptide form (DP group) or free amino acids (FAA group). The third was not supplemented with lysine (LF group). The fourth group of fish was fed commercial starter Bio-Oregon (C group). Histological or histomorphometric analyses were conducted: white muscle area, the total number of muscle fibers, the total number of white muscle nuclei, muscle fiber area, number of proliferating myonuclei. Fish fed LF diet showed the lowest number of nuclei and satellite cells proliferation. Results in DP and FAA groups were similar to that observed in fish fed C diet. Summarizing, wheat gluten-based diets supplemented with Lys-Gly dipeptide or free Lys and Gly amino acids exert beneficial effects on the morphology of yellow perch white muscle.

## 1. Introduction

Yellow perch (*Perca flavescens*) is a native percid species specific to waters of North America. Like all other species of perch, yellow perch is a very desirable consumption and sport fish. Moreover, the supply of perch meat does not meet the market demands, which is also one of the reasons for the dynamical development of the percid aquaculture all over the world [1,2]. Natural resources of which fishmeal and fish oil are obtained are limited. Due to the constant development of aquaculture, it seems necessary to look for alternative sources of protein that could be used in fish feed production. Plant proteins such as soybean meal or wheat gluten (WG) seem to be the basic source of protein [3]. Diets containing plant protein are cheaper and their nutritional values often do not differ from diets based on fishmeal. Wheat gluten is a promising source of well-digestible and high-quality plant protein; however, the amino acid composition of WG is characterized by the low content of lysine [4]. Therefore, in the production of feeds based on this protein source, lysine supplementation is necessary. Besides the low amount of lysine, WG has a well-balanced amino acid profile rich in sulfur amino acids. Moreover, WG is also distinguished by its high content of glutenin which is particularly important because of the noticeable improvement in digestibility of diet and stimulation of the fish immune system [4]. According to Helland and Grisdale-Helland (2006) [5], WG is a good substitute for fishmeal as a protein source for fish fed, and its content in the diet can reach up to 30% of total protein. The use of this alternative source of protein supplemented with deficient amino acids (e.g., lysine) has been proved in aquaculture of many fish species such as rainbow trout (*Oncorhynchus mykiss*) [6,7,8], cod (*Gadus morhua*) [9], common carp (*Cyprinus carpio*) [10,11], Atlantic salmon (*Salmo salar*) [12], or yellow perch (*Perca flavescens*) [13]. Hitherto, research was focused mainly on the wheat gluten-based diet effect on fish growth and morphology of the gastrointestinal tract [6,7,10]. The development of fish muscle tissue during feeding wheat gluten-based diets is studied only in common carp [14]. Many authors suggest that environmental factors and nutrition are the main factors that can significantly affect the development and growth of muscle [14,15]. The process of yellow perch muscle tissue development is not well known, but due to the rapid increase of interest in percids aquaculture, including Europe [16,17], investigation of this issue seems necessary.

Therefore, this study aimed to investigate the effect of diets based on wheat gluten supplemented with free amino acids Lys and Gly or Lys-Gly dipeptide on the white muscle morphology of yellow perch.

## 2. Materials and Methods

The experiment was performed in the Aquaculture Laboratory at the School of Environment and Natural Resources, Ohio State University, USA. This protocol was evaluated and approved by the 3rd Warsaw Local Ethics Committee for Animal Experimentation at the Warsaw University of Life Sciences (No. 28/2006, 29.06.2006, III`rd Local Ethics Committee, Warsaw). Yellow perch (*Perca flavescens*) of the initial weight of approximately 0.3 g were randomly allocated in 12 tanks (30 L), 60 fish per tank. The fish were fed three types of diets (in triplicates) based on WG: enriched with Lys-Gly dipeptide (DP group), enriched with Lys and Gly amino acids (FAA group), without lysine supplementation (LF group) (Table 1 and Table 2). The fourth experimental group was fed commercial starter Bio-Oregon (Westbrook, Maine, USA, C group) (in triplicates). The protein content in the diets formulated based on WG was 460 g/kg (DP, FAA, and LF groups), while in the commercial diet—435 g/kg (C group). The lipid content in the DP, FAA, and LF diets was 60 g/kg, while in C diet—155 g/kg [13]. The proximal composition of experimental diets is presented in Table 2. During the experiment, fish were fed manually, 3 times a day for 48 days. For the first 14 days of the experiment, the feed ration was 3% of fish biomass, while in the following period it was raised to 4–5% (up to the 48th day of the experiment).

On the last day of the experiment, all fish from each tank were weighed. For histological analysis, 15 fish from each experimental group (5 fish from each tank) were randomly collected. The fish were anesthetized with MS-222 (tricaine methanesulfonate, 3-amino benzoic acid ethyl ester, Sigma-Aldrich, St. Louis, MO, USA) and fixed in 4% buffered formalin (whole fish). Collected samples were dehydrated in series of ethanol (50%, 70%, 80%, 90%, 96%, 99.8%), cleared in xylene, and embedded in paraffin and cross-sectioned to 5 µm thickness sections at the height of the anus, according to the method described by Ostaszewska et al. (2008) [22]. Obtained histological sections were stained using the standard procedure of hematoxylin-eosin (H/E) (Avantor Performance Materials Poland S.A., Gliwice, Poland). To visualize the proliferating cells (satellite cell markers), immunohistochemical detection of Proliferating Cell Nuclear Antigen (PCNA) was used. The histological slides were deparaffinized in xylene and rehydrated in a gradient of ethanol. Endogenous peroxidase was blocked using 3% hydrogen peroxide. The slides were rinsed in Tris buffer (pH 8.0, Sigma-Aldrich, St. Louis, MO, USA) and incubated with monoclonal mouse primary antibody anti-PCNA, clone PC10 (cat no. M0879; DAKO, Glostrup, Denmark) for 1 h at room temperature. The visualization was performed using DAKO EnVision + System—HRP (DAKO, Glostrup, Denmark) according to the manufacturer’s instruction. Cell nuclei were counterstained using Harris hematoxylin. Then, slides were dehydrated, rinsed in xylene, and mounted in DPX (Sigma-Aldrich, St. Louis, MO, USA). For the negative control of the reaction, the slides without antibody incubation were used. Microscopic observations were performed using the Nikon Eclipse 90i microscope and the photos were taken using Nikon Digital Sight DS–U1 camera (Nikon Corporation, Tokyo, Japan). Histomorphometric measurements were made using the NIS—Elements AR 2.10 software (Nikon Corporation, Tokyo, Japan).

Histomorphometric measurements were conducted on the upper right quarter of the cross-section at the level of the anal opening (five sections per fish) and included: white muscles area (CSAw), the total number of muscle fibers (TFN), the total number of white muscle nuclei (TNN), muscle fiber area (FA), and number of the PCNA-positive myonuclei on the epaxial quarter of white muscle. Moreover, to estimate hypertrophy and hyperplasia processes, the following parameters were calculated: TFN/CSAw—index of muscle hypertrophy, TNN/TFN—index of number of muscle fiber nuclei, PCNA/TNN—index of proliferation nuclei [23,24].

The statistical processing of the obtained results was performed using the Statistica 12.0 software (StatSoft Inc., OK, USA). The mean and standard error of the mean for histomorphometry measurements were calculated for all groups. Statistical analysis was performed using a one-way analysis of variance (ANOVA) followed by Tukey’s post hoc test (*p* ≤ 0.05). Before the ANOVA analysis was performed, its assumptions were checked: normality (Shapiro–Wilk’s test) and variance homogeneity (Levene’s test).

## 3. Results

After the 48-day feeding period, the bodyweight of fish did not differ regardless of the type of used feed (*p* ≤ 0.05), the mortality of fish during the experiment was not observed, see in [13].

**Figure 1:**
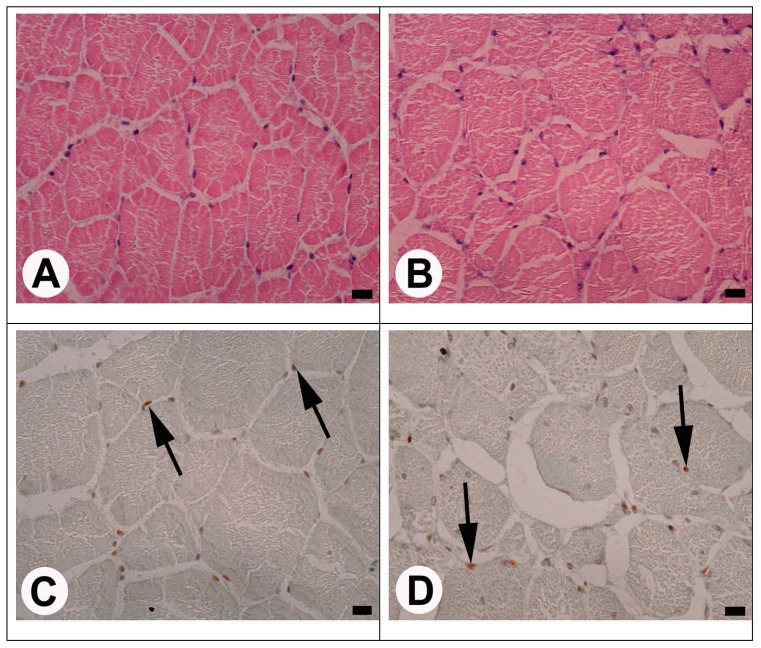
The cross-section of yellow perch white muscle ((**A**) and (**B**)—hematoxylin-eosin staining) and immunohistochemical detection of the proliferating cell nuclear antigen (PCNA) -positive myonuclei ((**C**) and (**D**); **arrows**) in this muscle after 48-day feeding with experimental diets based on wheat gluten with additional supplementation of Lys-Gly dipeptide ((**A**) and (**C**), respectively) or without lysine supplementation ((**B**) and (**D**), respectively). Scale bars = 10 μm.

The histomorphological analysis showed the correct structure (without histopathological changes such as lymphocyte infiltration, adipocyte deposition, connective tissue hypertrophy) of the white muscle of fish fed DP, FAA, and C diets. Although histological analysis of the cross-sections of samples from all groups showed the mosaic growth of muscle (Figure 1), in muscles collected from fish fed the LF diet, wide empty spaces within the fibers were observed. These spaces were wider compared to another group which may suggest, for example, the deposition of adipocytes in white muscles. These observations suggest the beginning of muscular degeneration or delayed myofibril synthesis (Figure 1).

After the experimental feeding period, on the last day of the experiment, the largest area occupied by white muscles was observed in the fish fed the FAA diet, whereas the smallest in fish fed the LF diet, but did not differ between groups (*p* > 0.05) (Table 3). The largest value of TFN was found in the fish fed DP and FAA diets, while the smallest was observed in the group fed the LF diet group. The TFN values of the fish fed LF group were significantly lower than in other groups (*p* ≤ 0.05) (Table 3).

The highest value of TFN was found in fish fed the DP and FAA diets, while the lowest was observed in the group fed the LF diet group, the differences were statistically significant (*p* ≤ 0.05) (Table 3). The largest muscle FA was found in fish fed the FAA diet, whereas the smallest in fish fed the C diet. The highest TNN value was observed in fish fed the FAA diet while the lowest in fish fed the LF diet (Table 3). No statistical difference was found in FA and TNN (*p* > 0.05) (Table 3).

On the last day of the experiment, the largest number of PCNA—positive myonuclei was found in fish fed FAA compared with other groups, while the least number was observed in fish fed the C diet (*p* ≤ 0.05) (Figure 1, Table 3).

No statistically significant differences were found analyzing the index of muscle hypertrophy and the index of proliferation nuclei between groups. The lowest value of the index of number of muscle fiber nuclei was observed in the DP group (Table 3).

## 4. Discussion

Muscle tissue in the body composition of fish is species-dependent and ranges from 40% to 60% of body mass. Therefore, its growth and development is a crucial factor in the context of aquaculture research. The growth of fish muscle in contrast to most vertebrates comprises two processes—hyperplasia and hypertrophy [25,26]. The hyperplastic growth of muscle tissue may be divided into two stages. Stratified hyperplasia is the miogenetic stage that occurs during embryogenesis and shortly in the post-hatching period [27] and during which the main muscle layers are forming [28]. During the next stage, mosaic hypertrophy, an increase in the number of muscle fibers is observed [28]. The mosaic hypertrophy is characterized also by significant differentiation of the muscle fibers area [29], which can be observed in histological sections. Hypertrophic muscle growth results in the larger area of muscle fibers as their diameter increases [30]. The balance between hypertrophy and hyperplasia is dependent on many factors, both genetic and environmental, among which feeding seems to be the crucial factor affecting the development and growth of fish muscle [14,22]. The results obtained in the present study showed that WG may be considered as a valuable alternative source of protein in the diet for aquaculture of yellow perch. However, it is generally known that WG used as a fishmeal substitute should be supplemented with lysine [4]. According to Kamaszewski et al. (2014) [14], supplementation of the diet with lysine in the free or dipeptide amino acid form had a positive impact on the growth and development of common carp white muscle. Similarly, Ostaszewska et al. (2013) [13] confirmed that both forms of lysine supplementation are suitable for optimum growth and development of yellow perch juvenile stages. The histological analysis conducted in the present study showed the positive effect of FAA and DP diets on white muscle morphology, more precisely, on the diameter of white muscles and the number of the PCNA positive myonuclei, which reflect the ability of muscle cells to proliferate. It was confirmed that the PCNA protein indicated the replication or repair processes of myonuclei [31]. Similarly, common carp feeding with feeds based on WG supplemented with free Lys and Gly amino acids had a beneficial effect on the proliferation of muscle cells, whereas fish feeding the WG-based diet supplemented with Lys-Gly dipeptide stimulated the development of muscle tissue by the increase of TFN [14]. Not one parameter of fish feed with FL and DP diets differed significantly from those obtained in fish fed the commercial C diet. Such results confirmed that the replacement of fishmeal with a plant protein source may have a positive effect on the metabolism of muscle cells, which may be caused by the presence of antioxidants, antiviral, and anti-inflammatory compounds in WG [32,33,34]. On the other hand, the results obtained in the group fed the LF diet showed the significantly lowest values of parameters TFN, the number of PCNA positive myonuclei, and a certain trend toward significant lowest values of CSAw and TNN. The low values of tested parameters such as CSAw, TFN, FA, and morphological changes of yellow perch muscle tissue fed the LF diet point to adverse changes in the muscle of fish. Most likely, these results are related to the deficiency of the indispensable amino acids and especially to the lack of lysine amino acid. Lysine is one of the growth limiting amino acids in fish [35]. Kamaszewski et al. (2014) [14] observed similar results in the experiment conducted on common carp fed the WG-based diet without the lysine supplementation. The aforementioned authors found the low values of TFN and FA on the muscle cross-sections compared to a fish fed a diet supplemented with Lys-Gly dipeptide or with the free form of these amino acids [14]. The obtained results of the index of muscle hypertrophy indicate that in the studied yellow perches, the process of muscle cell hypertrophy is not the main pathway for the development of muscle tissue. The dominant process is hyperplasia as evidenced by the results obtained when estimating the index of the number of muscle fiber nuclei. A lower value of this index was observed in fish fed DP, which indicates lower hyperplasia in fish in this group, however, there were no statistically significant differences in the value of the index of proliferation nuclei. This may suggest that the examined juvenile yellow perch are at the stage of hyperplastic growth of the white muscle tissue. This process is only slightly dependent on food intake as indicated by obtained results. Similar to results obtained by Kamaszewski et al. [14], it was confirmed the influence of the form of lysine availability or its lack on the recruitment rate of new muscle fibers, however, this phenomenon requires further research. The results obtained in the present experiment confirmed that the lysine is an essential amino acid, which affects growth [36] and muscle development [37] of fish.

## 5. Conclusions

The results indicate that the rearing of predatory species such as yellow perch, with the use of formulated feed based on plant protein (wheat gluten), is possible. Moreover, a fish fed a diet based on wheat gluten compared to commercial feed did not have statistically significant differences in body weight and mortality. Histological analysis of white muscles showed that plant protein supplemented with lysine, preferably in the dipeptide Lys-Gly form or free amino acids Lys and Gly, has a positive effect on their morphology and growth. There were no histopathological changes or lower values of TFN or PCNA positive myonuclei in fish fed these feeds compared to fish fed a diet without lysine supplementation. Based on the results and experience of previously published reports, it can be concluded that the use of the wheat gluten-based diet supplemented with lysine in dipeptide Lys-Gly or free amino acids Lys and Gly stimulate fish growth and development. Furthermore, feeds formulated based on wheat gluten are cheaper compared to traditional feed containing a high content of fish meal in composition. This is important as a consequence of the implementation of sustainable and nature-friendly aquaculture through the protection of natural resources (protection of schools of fish being the source of fishmeal) and the more effective use of agricultural raw materials in aquaculture.

## Figures and Tables

**Table 1:** The composition of the experimental diets (modified based on [13]).

Ingredients, g/kg Feed	Experimental Feeds		
	Supplemented with Lys-Gly Dipeptide (DP)	Supplemented with Free Lys and Gly Amino Acids (FAA)	Without Lys Amino Acid Supplementation (LF)
Fish meal	124	124	124
Wheat gluten (WG) ^a^	370	370	370
Wheat	172	172	172
Fish oil	50	50	50
Lecithin	150	150	150
Mineral mix	30	30	30
Vitamin mix	40	40	40
Lys-Gly Dipeptide ^a^	34	-	-
Lysine ^b^	-	21	-
Glycine ^b^	-	13	13
Glutamate ^d^	-	-	21
Cysteine ^c^	2	2	2
Arginine	6.5	6.5	6.5
Methionine	3.3	3.3	3.3
Threonine	2.9	2.9	2.9
Calcium monophosphate	15	15	15
Vitamin C	0.5	0.5	0.5

^a^ MP Biomedicals, Solon, OH, USA; ^b^ Bachem, Torrence, CA, USA; ^c^ [18]; ^d^ non-toxic level of glutamic acid [19].

**Table 2:** Proximate composition of diets used in the experiment [13,20,21].

Parametres (%)	Diets			
	DP	FAA	LF	C
Crude protein	46	46	46	43,5
Crude lipids	6	6	6	24
Ash	7	7	7	12
Moisture	8	8	8	8.5
Gross Energy (MJ/kg)	19.6	19.6	19.6	19.7

**Table 3:** Fish body weight and histomorphometric analysis of white muscle after 48-day feeding with experimental diets based on wheat gluten with additional supplementation of Lys-Gly dipeptide (DP) or free Lys and Gly amino acids (FL) or without lysine supplementation (LF), and commercial diet (C).

Parameters	Feed Group				
	DP	FAA	LF	C	*p* Value
Body weight, g	0.91 ± 0.02	0.90 ± 0.02	0.83 ± 0.01	0.79 ± 0.02	
CSAw	2557.1 ± 140.6	2873.5 ± 216.6	2032.6 ± 57.9	2701.2 ± 236.4	
TFN	797.7 ± 26.0 ^a^	768.0 ± 54.6 ^a^	515.3 ± 2.8 ^b^	679.3 ± 28.8 ^a,b^	DP vs. LF *p* = 0.0160 FAA vs. LF *p* = 0.0328
FA	1433.1 ± 12.7	1462.6 ± 52.2	1321.4 ± 20.2	1158.5 ± 80	
TNN	1424.3 ± 56.9	1590.0 ± 109.8	1049.0 ± 1.7	1392.3 ± 95.1	
PCNA—positive myonuclei	349.7 ± 5.3 ^b^	416.0 ± 17.2 ^a^	297.0 ± 20.8 ^b^	324.7 ± 4.8 ^a,b^	FAA vs. LF *p* = 0.0150
TFN/CSAw	3.17 × 10^−4^ ± 9.8 × 10^−6^	2.68 × 10^−4^ ± 1.6 × 10^−6^	2.56 × 10^−4^ ± 6.2 × 10^−6^	2.66 × 10^−4^ ± 1.4 × 10^−5^	
TNN/TFN	1.78 ± 0,02	2.07 ± 0.01 ^a^	2.036 ± 0.01 ^a^	2.02 ± 0.5 ^a^	DP vs. FAA *p* = 0.0045 DP vs. LF *p* = 0.0129 DP vs. C *p* = 0.0187
PCNA/TNN	0.25 ± 0.01	0.27 ± 0.01	0.28 ± 0.02	0.24 ± 0.01	

Measurements were taken on the upper right quarter of the cross-section included: white muscles area (CSAw) (mm^2^), the total number of muscle fibers (TFN) (number), the total number of white muscle nuclei (TNN) (number), muscle fiber area (FA) (μm^2^), and number of the PCNA-positive myonuclei on the epaxial quarter of white muscle (number), TFN/CSAw—index of muscle hypertrophy, TNN/TFN—index of number of muscle fiber nuclei, PCNA/TNN—index of proliferation nuclei. The data are presented as mean ± standard error of the mean (SEM), values with different superscripts (^a,b^) within each row differ significantly at *p* ≤ 0.05 according to Tukey’s post-hoc test (*n* = 15), *p* values of statistically significant changes between groups are presented in the *p* value column.
